# The scale and structure of government financial investment in traditional medicine based on optimal efficiency: evidence from public traditional Chinese medicine hospitals (PTHs) of Henan province, China

**DOI:** 10.1186/s12913-021-06185-x

**Published:** 2021-02-26

**Authors:** Weicun Ren, Xiaoli Fu, Clifford Silver Tarimo, Maisa Kasanga, Yanqing Wang, Jian Wu

**Affiliations:** 1 College of Public Health, Zhengzhou University, 100 Science Avenue Gaoxin District, Zhengzhou, People’s Republic of China; 2 College of Sanquan, Xinxiang Medical University, Xinxiang, People’s Republic of China; 3 Department of Science and Laboratory Technology, Dares Salaam Institute of Technology, Dar es Salaam, Tanzania; 4 University Teaching Hospital, Lusaka, Zambia

**Keywords:** Government, Financial investment, TCM hospitals, Revenue

## Abstract

**Background:**

Traditional medicine has been widely used to address relatively common illnesses. In this regard, Chinese government has been continually topping up its investments on public Traditional Chinese Medicine hospitals (PTHs) in recent years. This study aimed to assess the optimal scales and structure of the investments in Henan province by analyzing the contribution of Government Financial Investment (GFI) to the efficiency and revenue growth of PTHs as well as recommending proper investment strategies for implementation to policy-makers.

**Methods:**

This study was a panel data study, conducted in Henan Province, China. By collecting 143 PTHs’ operational data from the year 2005 to 2017, Barro Economic Growth (BEG) model, Stochastic Frontier Analysis (SFA) and Vector Autoregressive (VAR) model were used to assess the efficiency and PTHs revenue.

**Results:**

The study observed the positive contribution of GFI to PTHs’ revenue growth (average MPG = 2.84), indicating that the GFI had not reached the required optimal level of “Barro Law”. In order to maximize the input-output efficiency, the scales of GFI on Grade III, Grade II A, Grade II B PTHs need to be increased by − 5.96, 4.88 and 11.51%, respectively. The third year following the first investment may be a more essential period for conducting an effective GFI evaluation in Henan Province.

**Conclusions:**

GFI on PTHs usually has a long-term impact on PTHs. Governments can adjust its GFI policy so as to maximize the input-output efficiency.

## Background

Globally, traditional medicine has long been used to address relatively common illnesses [1]. Traditional Chinese Medicine (TCM) is widely accepted in China as a complement and alternative for medical treatment [2–5]. At present, TCM is mainly provided by public TCM hospitals (PTHs), private TCM hospitals and private clinics. But the contemporary private sectors which are the primary traditional medicine providers, appear to seek immediate cash payments that have frequently led to considerable debt and asset sales by traditional medicine users [6]. As a “main provider of last resort”, PTHs need to be further emphasized and developed. In addition to external factors such as modern medicine, TCM’s development is mainly affected by the internal influence of funds. And government investment intervention is necessary if PTHs want to survive and continue fulfilling their mission [7].

Previous researches suggest that the support of GFI can affect the progress as well as normal functioning of hospitals [8, 9]. Woolhandler et al. found that when experiencing severe cutbacks in funding for public medical facilities, the census at the county hospital declined by 65% while the number of the outpatient visits decreased by 50% [8]. Similarly and Shonick observed a sharp increase in both government health expenditures and hospital revenues while hospitals received government subsidies amounting to at least 60% of their total revenues [9].

In the past 10 years in China, the total government financial investment (GFI) in PTHs had reached 192.83 billion yuan to achieve the expected target of basic access to TCM services for everyone, and it’s currently mainly used for the improvement of infrastructure, maintenance of medical equipment and remuneration. Henan Province is an agricultural and emerging industrial province with over 100 million people, a very typical and representative province in central China. In 2017, the number of PTHs in Henan province reached 257, accounting for more than 10% of the national total PTHs. The bed capacity was more than 63,100, and the overall quality of care was better than the national average. The GFI reached 1.60 billion yuan in PTHs, accounting for 6.08% of the total GFI of Henan Province [10, 11].

Efficiency analysis in health care industry has attracted significant interest [12, 13] due to escalating GFI and health care costs, especially in countries where public involvement in health care provision is high [14]. Improvement in efficiency may be one of the best approaches that can bend the cost curve without compromising quality of care as it can yield higher returns to fixed investment [15]. Compared with the least efficient hospitals, high-efficiency hospitals tend to have lower average costs, higher labor productivity, and higher profit margins [16].

Kerr found that there are opportunities for public policies to support improved efficiency in public hospitals in Australia [17]. Colomb et al. found that ownership, specialization and scale are determinants of all types of efficiency [18]. While Chen et al. found that the changes introduced into the health care sector in China during the last 30 years have improved the cost efficiency of the whole system, especially after the public subsidies and medical insurance reforms [19].

In order to correctly formulate GFI strategies, improve the efficiency of capital use, and ensure the basic sustainable development of TCM, this study analyzed the operational status of 143 PTHs in Henan Province from the year 2005 to 2017 by using the Barro Economic Growth (BEG) model to explore the scales and structure of PTHs’ GFI based on promoting hospital revenue growth. The current input-output efficiency-maximizing investment strategy of GFI to PTHs was tested by using the Stochastic Frontier Analysis (SFA). At the same time, the most appropriate evaluation period was also estimated based on the long-term impact trend of GFI on PTH’s revenue growth by using Vector Autoregressive (VAR) model.

## Methods

### Classification of public TCM institutions

Public TCM institutions with less than 100 beds in China generally refer to community health service centers as primary health care institutions. Institutions with 100–500 beds are set as Grade II hospitals while Grade III hospitals are the institutions with more than 500 beds (9) (Table 1). Each grade was divided into A, B and C levels depending on department set, staffing, management level, technical level, work quality and technical facilities [20].

**Table 1:** Classification of TCM institutions

Classification	Beds	Responsibilities	District
Grade I	<= 99	Provide prevention, medical, health care, and rehabilitation services	Single community
Grade II (A, B)	100–500	Provide comprehensive medical and health services and undertake certain teaching and research tasks	Multiple communities
Grade III	> = 501	Provide high-level specialist health services and perform higher education and research tasks	Several areas

### Data collection

Henan Province has a total of 143 PTHs, including 23 Class III, 96 Class II A and 34 Class II B. The medical services of PTHs covered a population of more than 100 million and the total annual number of medical treatments reaches 18 million [5]. The data which was used in this study includes: TCM practicing clinical workers, suitable TCM technologies implemented, GFI, revenue and value of fixed assets et al. of 143 PTHs. All the data was derived from the Henan Provincial Health Statistics Yearbook and the hospital financial year reports from the year 2005 to 2017. This study was submitted to Zhengzhou University Medical Ethics Committee and the need for ethical approval was waived.

### Data management and analysis

#### Variable index selection

Considering the basic characteristics of PTHs and avoiding the impact of strong relationship among indicators, the study selected variables based on importance and availability [21]. According to the requirements for the BEG and SFA models [14, 15, 18], this study selected indicators from seven aspects of revenue, assets, technology, financial investment, personnel, service provision and effect. These include: 1. The PTHs revenue (Revenue) which refers to the sum of hospital income and asset appreciation; 2. The value of fixed assets (Fixed Assets) which is the total value of the houses, equipment, and transportation tools owned by the hospital; 3. Number of suitable TCM technologies implemented (Technologies) defined as the number of suitable TCM technologies implemented and provided by the hospital; 4. Government financial investment (GFI) interpreted as the total amount of funds invested by the government for hospital infrastructure, equipment purchase, service development, and implementation of medical reform policies; 5. Number of TCM practicing clinical workers (Clinical Workers) referred to the total amount of clinical workers in the hospital who have the qualifications to practice with the TCM; 6. Number of visits: this is defined as the total number of outpatient visits recorded in the hospital each year; 7. TCM treatment improvement rate (Improvement Rate) which is interpreted as the proportion of patients who have received TCM treatment.

#### The scales and structure analysis method - Barro Economic Growth (BEG) model

BEG model is derived from the theory of endogenous economic growth, which introduces technology as a variable into an economic system and uses the endogenously of technology (the effect of technology on economic growth, which is related to other random disturbances, not completely fixed) to explain economic growth [22]. In addition, GFI is introduced into the BEG model, as it is believed that GFI is another important factor affecting economic growth, and the famous “Barro law” is deduced, which is when the marginal product of GFI (MPG) is equal to 1, the scale of GFI reaches the optimal level [23]. In this study, MPG refers to the amount of hospital revenue increased when GFI increases by one unit. The specific model equation is expressed as:

$$ \mathrm{Y}={AK}^{\alpha }{L}^{\beta }{G}^{\delta } $$

In formula:

- Y: Revenue;
- A: the technological progress rate (constant term coefficient);
- K: Fixed Assets;
- L: Clinical Workers;
- G: GFI;
- *α*: the regression coefficients of variables K, which is also the output elasticity (the percentage change in output caused by a predetermined percentage change of an input element when all other input elements remain unchanged) of assets in growth of Y, reflecting the contribution of variable K to the dependent variable Y;
- *β*: the regression coefficients of variables L, which is also the output elasticity of labor in growth of Y, reflecting the contribution of variable L to the dependent variable Y;
- *δ*: the regression coefficients of variables G, which is also the output elasticity of financial inputs in growth of Y, reflecting the contribution of variable G to the dependent variable Y.

The natural logarithm of the above formula can be obtained:

$$ \mathrm{L} nY=C+\alpha LnK+\beta LnL+\delta LnG+\varepsilon $$

Among them:

- C: constant term;
- *ε*: the residual term, refers to the error between hospital revenue estimated by the model and that in actual situation;
- LnY, LnK, LnL, LnG: the logarithm of each variable, indicating the respective growth rates.

In this study, the output elasticity of GFI to hospital revenue growth is δ. Following equation is used to get the MPG:

$$ MPG=\delta \frac{Y}{G} $$

When MPG = 0, GFI has no effect on hospital revenue; when 0 < MPG < 1, GFI can effectively promote hospital revenue growth, while the scale of GFI is too small; when MPG = 1, it indicates the scale of GFI reaches the optimal level; when MPG > 1, GFI can effectively promote hospital revenue growth, while the scale of GFI is too large [23].

#### Input-output efficiency analysis method-Stochastic Frontier Analysis (SFA)

SFA was first proposed by Aigner, Lovell and Schmidt (1977), and is widely used to estimate the impact of macro-environmental factors on efficiency [14]. This study uses Fried’s SFA theory based on an input-oriented traditional Data Envelopment Analysis (DEA) model which was proposed by Banker, Charnes and Cooper in 1984 to evaluate the relative effectiveness of the same type units based on multiple input indicators and multiple output indicators to explore the impact of macro-environmental factors (refers to GFI in this study) on hospital efficiency [18, 24].

Firstly, we use the Fixed Assets, Technologies and Clinical Workers as input factors while the number of visits, Revenue and Improvement Rate are used as output factors. GFI is used as macro-environmental factors. An input-oriented traditional DEA model is established to measure the overall input-output efficiency of PTHs [25, 26]. Secondly, SFA model is used to decompose the results of the DEA model, using slack variable S_ni_ (the difference between the actual production process and the highest efficiency) of each input including Fixed Assets, Technologies and Clinical Workers as dependent variables while macro-environmental factor variable as independent variable. SFA regression equation is established for each S_ni_ [27–31]:

$$ {\mathrm{S}}_{ni}={f}^n\left({Z}_{ni}\cdot {\beta}^n\right)+{v}_{ni}+{\mu}_{ni} $$

In the formula:

- Z_ni_: GFI (macro-environment variable);
- *β*^*n*^: parameter which is estimated by macro-environment variable;
- *f*^*n*^(*Z*_*i*_ ⋅ *β*^*n*^): the influence of macro-environmental variables to S_*ni*_;
- *v*_*ni*_: random interference term;
- *μ*_*ni*_: management level;

$$ n=1,2,\cdots, N,\kern0.5em i=1,2,\cdots, I. $$

#### Evaluation period selection method - Vector Autoregressive (VAR) model

VAR model is constructed to directly estimate the long-term relationship of multiple variables, and to construct multiple joint equations on this basis. In order to obtain the best regression results without bias, the model requires the data to be stable or there is a co-integration relationship between variables. In this study, the stable refers to mean and variance of all variables have no systematic and periodic changes and the co-integration relationship refers to change trend of two and more variables is linearly related. Augmented Dickey-Fuller (ADF) test method is used to test the stability of data. If the test result shows that one variable is non-stationary, differential transformation (approximate the derivative by finite difference to find the approximate solution of the equation) is usually performed on the data, and then test is repeated until the variable is stable. This study chooses Johansen cointegration test (including tracking test and the λ-Max statistic tests) which is often used to test the relationship between two or more variables, to test whether there is a co-integration relationship between variables.

Based on the results of BEG model, VAR model is used to estimate the most appropriate evaluation period of GFI by analyzing the long-term impact trend of Revenue and GFI on others or themselves. By establishing the impulse response function that is commonly used to analyze the present and future effects of random changes imposed on the endogenous variable on itself and other variables, the long-term impacts of short-term change in GFI on PTHs’ GFI and Revenue are analyzed, characterizing the response for the variables to specific change.

### Analysis tool

The initial data was collected and processed by using Excel 2016. DEA and SFA analysis were performed by using DEAP 2.1 and Frontier 4.1 software, respectively. BEG and VAR were performed by using Eviews6.0 software. *P* <  0.05 was considered statistically significant.

## Results

### Operational information of PTHs

Between 2005 and 2017, the number of clinical workers from 143 PTHs in Henan Province increased from 32,058 to 64,669, with an average annual growth rate of 6.02% while the Fixed Assets increased with an average annual growth rate of 15.61%. The Revenue increased from 1.83 billion yuan to 16.37 billion yuan, with an average annual growth rate of 20.02%. While the GFI increased from 0.14 billion yuan to 1.61 billion yuan, with an average annual growth rate of 22.97% (Table 2).

**Table 2:** The operational information of sample PTHs

Year	Clinical Workers^a^ (10,000 person)	Fixed Assets^b^ (billion yuan)	Revenue^c^ (billion yuan)	GFI^d^ (100 million yuan)	GFI/Revenue (%)
2005	3.21	2.33	1.83	0.14	7.65
2006	3.34	2.64	1.99	0.16	8.04
2007	3.27	2.56	2.45	0.20	8.16
2008	3.49	2.95	3.23	0.27	8.36
2009	3.70	3.57	4.01	0.45	11.22
2010	3.82	4.10	4.93	0.68	13.79
2011	4.31	5.10	6.35	0.67	10.55
2012	4.62	6.22	8.38	0.61	7.28
2013	5.04	7.48	9.96	0.78	7.83
2014	5.68	9.04	12.28	0.90	7.33
2015	6.12	10.33	13.37	1.19	8.90
2016	6.42	12.09	14.55	1.54	10.58
2017	6.47	13.26	16.37	1.61	9.84
Average annual growth rate (%)	6.02	15.61	20.02	22.97	9.20

^a^Clinical Workers stands for Number of TCM practicing clinical workers

^b^Fixed Assets stands for value of fixed assets

^c^Revenue stands for the revenue of public TCM hospitals

^d^GFI stands for governmental financial investment

Table 3 displays the descriptive statistics of Grade II A, Grade II B and Grade III PTHs in 2017. In overall, Grade III PTHs had better economic indicators than other hospitals, with an average GFI, Revenue, Fixed Assets of 238 million, 4.00 billion and 1.99 billion yuan, respectively. Grade III, Grade II A and Grade II B PTHs had 1146, 361 and 150 Clinical Workers, respectively. The average number of visits in the three types of hospitals were 0.64, 0.17, and 0.10 million. The number of suitable TCM technologies implemented in Grade III PTHs were 26, while Grade II A and Grade II B PTHs were both 17. The average Improvement rate of the three types of PTHs exceeded 88%, but the Improvement Rate of one Grade II A PTHs were only 40.12%.

**Table 3:** Characteristics of PTHs’ efficiency evaluation indexes

Hospital grade	Sample size	Indicators	Input indicators			Output indicators			Macro-en-vironmental factor
			Fixed Assets	Clinical Workers	Technol-ogies	Number of visits	Revenue	Improvement Rate	GFI
			billion yuan	100 persons	items	million times	billion yuan	percentage	100 million yuan
Grade III	23	Max^a^	4.45	21.25	40.00	2.39	16.72	99.90	5.16
		Min^b^	0.97	5.75	10.00	0.89	6.85	74.90	0.22
		AVG^c^	1.99	11.46	25.70	0.64	4.00	94.07	2.38
		SD^d^	1.311	6.95	12.54	0.48	3.39	6.50	2.45
Grade II A	96	Max	3.29	8.25	33.00	0.57	3.39	100	3.08
		Min	0.53	0.75	9.00	0.13	0.61	40.12	0.05
		AVG	0.80	3.61	17.26	0.17	0.77	91.08	0.92
		SD	0.37	1.75	14.57	0.11	0.49	10.91	1.02
Grade II B	24	Max	1.06	2.90	20.00	0.24	0.88	98.50	2.98
		Min	0.32	0.60	8.00	0.05	0.15	63.00	0.19
		AVG	0.42	1.50	17.22	0.10	0.30	88.45	0.76
		SD	0.18	0.70	13.29	0.06	0.18	8.52	0.85

^a^*Max* Stands for Maximum

^b^*Min* Stands for Minimum

^c^*AVG* Stands for Average

^d^*SD* Stands for Standard deviation

### Scales and structure analysis of PTHs’ GFI based on revenue promotion

#### Indicator selection and feasibility test

Results from the ADF model showed that the statistical results of each variable were smaller than critical results (0.05) on the second-order difference (D2), indicating that all four variables were second-order single-integer sequences (Table 4). It means the data becomes stationary series after the second difference. And at the 5% significance level, Johansen test method revealed that there were at most two co-integration relationships between variables (*P* <  0.05) (Table 5). This indicated that the linear combination of four variables had long-term stability, and the model could be further analyzed.

**Table 4:** Unit root test results of variables (0.05)

Variable	Level test result		First-order difference result (D)		Second-order difference result (D2)	
	ADF result	Critical result	ADF result	Critical result	ADF result	Critical result
LN Y	1.7439	−1.9777	−0.4981	−1.9777	−3.9300	−1.9823
LN K	7.6575	−1.9740	−0.1238	−1.9823	−5.9347	−1.9823
LN L	4.9011	− 1.9740	− 1.2430	− 1.9777	−5.2242	− 1.9823
LN G	4.0416	− 1.9740	− 1.2776	− 1.9777	−2.8411	− 1.9823

**Table 5:** Co-integration test results between multiple variables

Original hypothesis	Eigenvalue	Trace (*P*)	λ-Max Eigenvalue (*P*)
No co-integration vector	1.0000	338.3943 (0.0001)	267.6401 (0.0001)
At most one co-integration variable	0.9870	70.7542 (< 0.0001)	47.7566 (< 0.0001)
At most two co-integration variable	0.7941	22.9976 (0.0006)	17.389 (0.0037)
At most three co-integration variable	0.3998	5.6147 (0.0212)	5.6147 (0.0212)

#### Scales and structure analysis

In this study, the regression analysis was performed on the model by Ordinary Least Squares method that is most commonly used to solve curve fitting problems. It can easily obtain unknown data and minimize the sum of squared errors between obtained data and actual data. The regression results were as follows:

$$ {\displaystyle \begin{array}{c}\mathrm{Ln}\ \mathrm{Y}=-1.4433+1.5218\mathrm{LnK}-1.4157\mathrm{LnL}+0.2503\mathrm{LnG}\\ {}\left(-0.7498\right)\kern0.5em (1.7453)\kern0.5em \left(-0.8210\right)\kern0.5em (1.6261)\\ {}\begin{array}{cc}{\mathrm{R}}^2=0.9858& \mathrm{DW}=1.0740\end{array}\end{array}} $$

It could be seen from the above regression results that the result of model’s fitting index R^2^ was 0.99, indicating the goodness of fit of the model as well as significant linear relationship among variables. And the hypothesis that MPG = δY/G could be obtained as MPG = 0.25Y/G. The average result of Y/G in TPHs from the year 2005 to 2017 was 11.35, making an average MPG to be 2.84 (the minimum result was 1.82 and the maximum result was 3.46).

GFI scales of PTHs had not yet reached the optimal level as per “Barro Law” which requires MPG result to be 1. In addition, from MPG > 0, it could be seen that the GFI had a positive economic impact on promoting the growth of PTHs’ revenue.

### Efficiency-maximizing investment strategy of GFI on PTHs

#### Input-output efficiency analysis

In the calculation results of SFA model, management level factor (*μ*_*ni*_) was found to be the main attribute affecting Fixed Assets and Clinical Workers (*γ ≈ 1, P <  0.05*). And in Grade III PTHs, the difference between the management level factor and the random interference term (*v*_*ni*_) had little impact on the efficiency, but it had an impact on the direction of efficiency (*γ ≈ 1, P > 0.05*). Further analysis found that, increasing the GFI will reduce the efficiency of Grade III PTHs (*β > 0, P <  0.05*), and it will improve the efficiency of Grade II B PTHs (*β < 0, P < 0.05*). In Grade II A PTHs, increasing the GFI will reduce the waste of Fixed Assets and Clinical Workers (*β < 0, P < 0.05*), but it will intensify the waste of Technologies (*β > 0, P < 0.05*). See Tables 6 and 7 for details.

**Table 6:** The impact of GIF on the efficiency of input factors

Independent variable	Fixed Assets			Clinical Workers			Technologies		
	Grade III	Grade II A	Grade II B	Grade III	Grade II A	Grade II B	Grade III	Grade II A	Grade II B
Constant term	0.56E+ 01***	0.22 + 01***	0.46E+ 01***	0.18E+ 01**	0.11E+ 01***	0.07E+ 01***	0.32E+ 01***	0.32E+ 01**	0.28E+ 01
	0.12E+ 01	0.66E+ 00	0.76E+ 00	0.86E+ 00	0.76E+ 00	0.10 E+ 00	0.82E+ 00	0.18E+ 01 0.19	0.19E+ 01
	0.30E+ 01	0.94E+ 01	0.86 E+ 01	0.24E+ 01	0.61 E+ 01	0.36E+ 02	0.39E+ 01	E+ 01	0.96E+ 00
GFI	0.32E+ 00***	− 0.63E-01**	-0.10E+ 00**	0.40E− 01***	-0.20E-01**	-0.41E-01***	0.79E− 01	0.90E-01**	-0.25E-01**
	0.52E− 01	0.49E-01	0.92 E-01	0.66E− 01	0.58E-01	0.80 E-02	0.69E-01	0.63E-01	0.13E-01
	0.60E+ 01	-0.13E+ 01	− 0.17E+ 01	0.61E+ 01	-0.35E+ 01	− 0.51E+ 01	0.89E+ 00	0.14E+ 01	− 0.19E+ 01
δ^2^	0.11E+ 00***	0.32E+ 00***	0.77E+ 00**	0.11E+ 01***	0.44E+ 00*	0.92E+ 00***	0.27E+ 00	0.67E+ 00***	0.45E+ 00***
	0.32E− 01	0.47E− 01	0.35E+ 00	0.44E+ 00	0.32E+ 00	0.92E− 01	0.25E+ 00	0.93E− 01	0.13E+ 00
	0.35E+ 01	0.68E+ 01	0.22E+ 01	0.25E+ 01	0.14E+ 01	0.10E+ 02	0.11E+ 01	0.72E+ 01	0.35E+ 01
γ	0.95E+ 00***	0.88E+ 00***	0.90E+ 00***	0.95E+ 00***	0.91E+ 00***	0.99E+ 00***	0.90E+ 00	0.99E+ 00***	0.87E+ 00**
	0.74E− 01	0.36E− 01	0.14E+ 00	0.85E− 01	0.61E− 01	0.20E− 05	0.51E-01	0.11E-04	0.18E-01
	0.13E+ 02	0.25E***	0.62E+ 01	0.64E+ 01	0.50E+ 01	0.50E+ 06	0.72E+ 01	0.95E+ 05	0.42E+ 01
Likelihood function absolute value	-0.75E+ 01	-0.81E+ 02	-0.20E+ 02	-0.13E+ 02	-0.82E+ 02	-0.16E+ 02	-0.22E+ 02	-0.12E+ 03	-0.24E+ 02
Unilateral error likelihood ratio test	0.74E+ 01	0.13E-01	0.89E+ 00	0.69E-01	0.77E-01	0.47E+ 01	0.16E+ 01	0.24E+ 01	\

*, **, *** indicate significant levels at 0.1, 0.05, and 0.01, respectively. The values below the horizontal line are test values, *P* values, variances, respectively

**Table 7:** Influence direction of GFI on PTHs input indicators (0.05)

Hospital grade	Fixed Assets	Clinical Workers	Technologies
Grade III	-^a^	–	–
Grade II A	+^b^	+	–
Grade II B	+	+	+

^a^“+” means that the increase in GFI will increase the efficiency of input utilization

^b^“—” means that the increase in GFI will increase the waste of input

#### The efficiency-maximizing investment strategy of GFI

Based on the results of Tables 6 and 7, this study explored the efficiency-maximizing investment strategy of GFI in three type of PTHs. We found that, in order to achieve maximum input-output efficiency, Grade III, Grade II A and Grade II B PTHs’ GFI need to increase by − 5148, 9263 and 2034 million-yuan, accounting for − 5.96, 4.88 and 11.51% of current amount, respectively. Similarly, the study also described the investment strategy of Fixed Assets and Clinical Workers corresponding to GFI (Table 8).

**Table 8:** The efficiency-maximizing investment strategy

Hospital grade	GFI	Fixed Assets		Clinical Workers	
	Increment^a^ (%^b^)	Increment (%)	Eliminate the increment after GFI impact (%^c^)	Increment (%)	Eliminate the increment after GFI impact (%)
	100 million	100 million yuan	100 million	100 persons	100 persons
Grade III	−51.48 (− 5.96)	5.26 (11.49)	4.58 (−1.49)	26.34 (9.99)	24.26 (−0.79)
Grade II A	92.63 (4.88)	9.99 (13.01)	13.23 (4.22)	41.55 (11.99)	47.44 (1.70)
Grade II B	20.34 (11.51)	1.28 (17.66)	2.49 (7.04)	5.28 (14.67)	6.84 (4.33)

^a^Increment refers to the amount of input required to maximize efficiency

^b^The ratio of the amount of input required to achieve maximum efficiency to the current amount

^c^Eliminate the ratio of inputs affected by GFI to the current amount

**Fig. 1:**
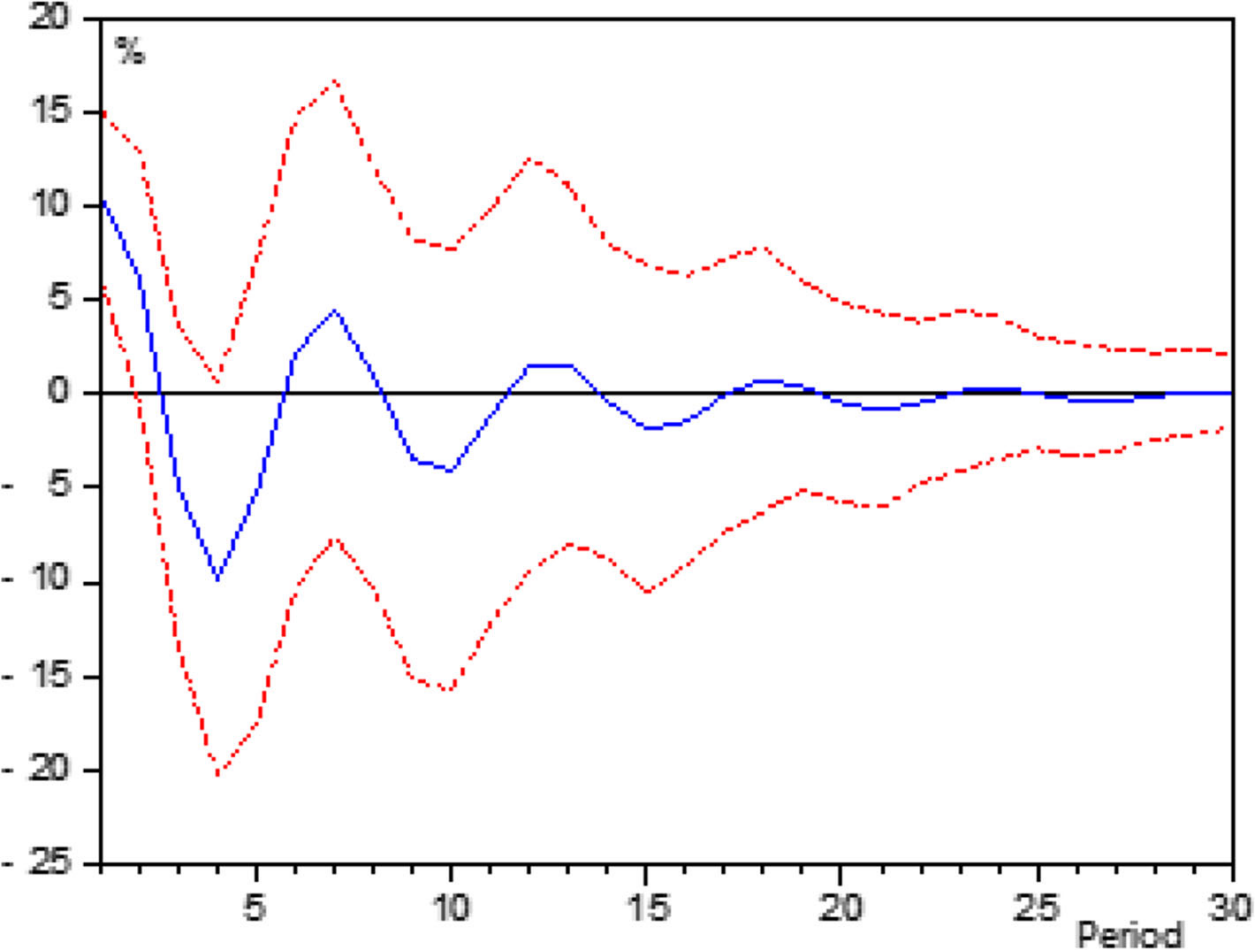
Impulse response of GFI to its own changes. The horizontal axis represents the duration period of the impulse response function, and the vertical axis represents the responsiveness of PTHs’ GFI to GIF changes. The solid line in the middle represented the impulse response function, and the dotted line represented the deviation from the standard deviation of two times

This study created a VAR model with four variables including: Revenue, Fixed Assets, Clinical Workers and GFI of PTHs, and analyzed the impulse response function. The results showed that the change of GFIs’ scale had a cyclical decrease trend in its long-term impact, and its overall cumulative impact was almost zero. Generally speaking, in China, the current changes in GFI will not affect the scale of future GFI, which makes it simple and feasible to adjust the current GFI according to the efficiency-maximizing investment strategy (Fig. 1).

### The most appropriate evaluation period of GFI

**Fig. 2:**
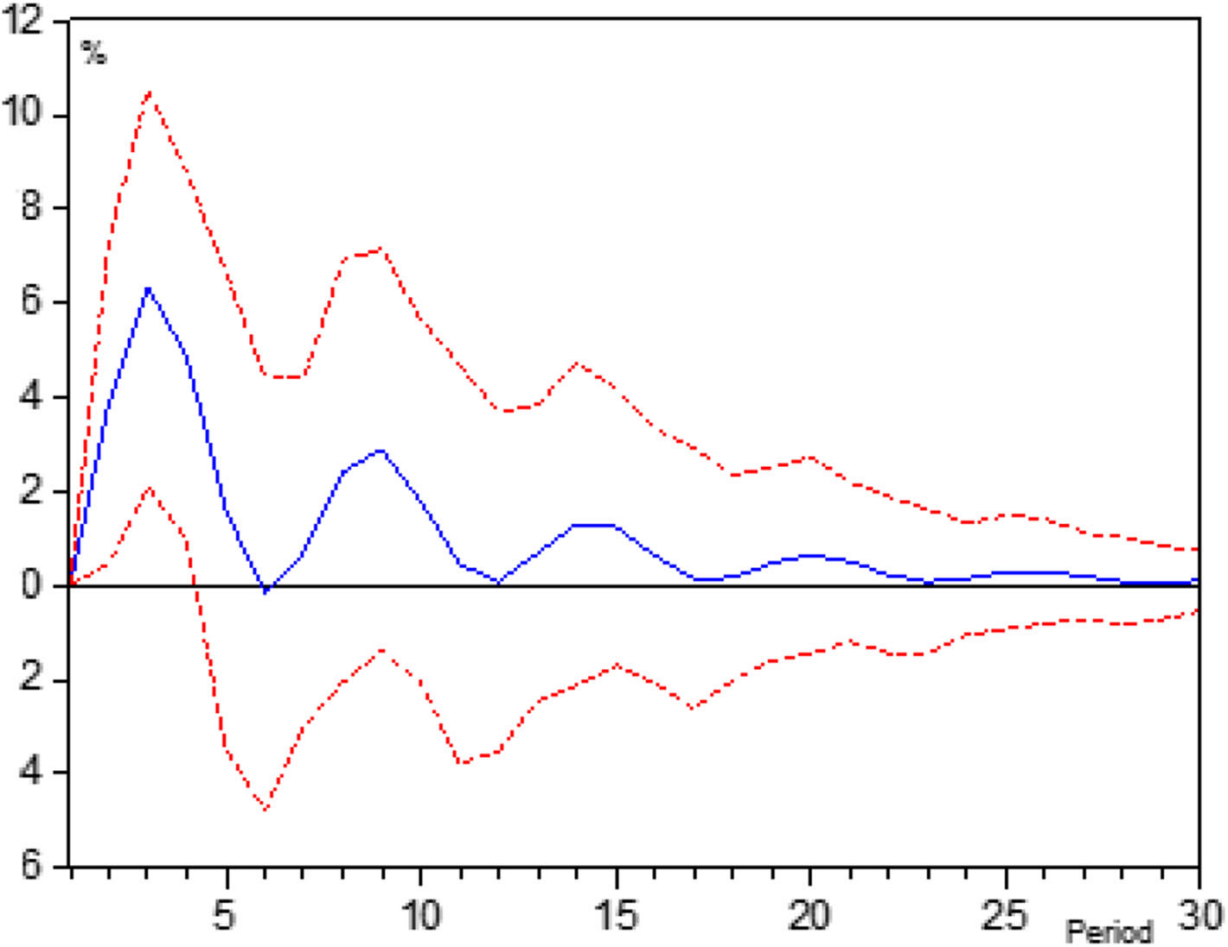
Impulse response of Revenue to GFI. The horizontal axis represents the duration period of the impulse response function, and the vertical axis represents the responsiveness of PTHs’ Revenue to GIF changes. The solid line in the middle represented the impulse response function, and the dotted line represented the deviation from the standard deviation of two times

The study also analyzed the most appropriate evaluation period based on the long-term impact trend of GFI on PTH’s revenue growth by using VAR model. As shown in Fig. 2, firstly, GIF had a positive impact on PTHs’ revenue, and the impact will gradually decline after the third period following the first year of investment (1 year as a period in PTHs). Secondly, this positive economic impact had completely disappeared after the 17th period. In short, the GFI had a significant role in promoting the revenue growth of PTHs, and the third year after the investment year may be a more effective period for conducting an effect evaluation of GFI in Henan Province.

## Discussions

This study aimed to assess the optimal scales and the structure of investments by analyzing the contribution of GFI to the efficiency and revenue growth of PTHs in Henan Province, China. The study found that MPG result of GFI to PTHs’ revenue was greater than 1 between 2005 and 2017 (the average MPG = 2.84), indicating the optimal GFI scales of MPG = 1 as per the “Barro Law” has not yet been attained. Schlesinger and colleagues found that the traditional medical providers were included in the medical aid waivers in order to help achieving the “Triple Aim” goals in the United States [32]. It is suggested that the government should carry out sustainable strategic design when arranging hospital investment, and allocating sufficient resources will help to effectively address patient needs, technological changes and increased awareness [33, 34]. The growth of Fixed Assets also has a positive impact on PTHs’ revenue growth [16]. And Fixed Assets have the greatest promotion impact (*β* = 1.5218) on PTH’s Revenue growth of the three variables of GFI, Fixed Assets and Clinical Workers. It shows that, if the PTHs’ GFI can be more applied to infrastructure construction and equipment purchase, it can promote the increase in PTHs’ revenue to a greater extent.

Previous studies have suggested that GFI has an impact on the efficiency of different-scale PTHs’ input-output, and the degree of impact is related to characteristics of hospital and market [35, 36]. This study found that increasing GFI would reduce the input-output efficiency of Grade III PTHs (5.96% reduction of the current amount to achieve maximum efficiency). For Grade II PTHs, increasing GFI would increase the efficiency of Fixed Assets and Clinical Workers. One likely reason for this phenomenon is that, large-scale and high-tech hospitals have sufficient funds, while hospitals with limited size and technology are limited in source channels, and rely more on government support [37]. Therefore, focusing the GFI on smaller traditional medicine hospitals will contribute to the development of traditional medicine [38].

Simultaneously, the impact of GFI on PTHs’ Revenue has been steadily weakening, lasting about 17 periods, and the overall impact was positive (sum of 17 periods’ impact > 0). If the impact on the current GFI is to be evaluated, the impact evaluation within a single investment period (1 year in China) is suitable for the next third period to obtain the maximum result and the overall impact evaluation should be conducted after the 17th period. Governments and hospitals should carefully formulate GFI impact evaluation plan and select appropriate evaluation cycles to accurately assess the effectiveness of GFI.

This study attempted to estimate the current state and the efficiency-maximizing investment strategy of PTH’s GFI from one of the most populated provinces of China. Findings from this study may help the government in formulating an effective GFI strategy and hence promoting the development of PTHs. However, there are still some limitations in this study. We recommend further studies to classify hospitals based on hospital bed capacity rather than the official hospital classification criteria. In addition, the time span for data collection should be further extended so as to improve the accuracy and credibility of the results. Furthermore, there is also a room for studies on optimization in the selection of indicators.

## Conclusions

The GFI for TCM has experienced a period of rapid growth over the past 12 years, but we found no strong evidence that its actual impact is consistent with the expected target of achieving basic access to TCM services for everyone. Although our analysis was limited to PTHs, it provided important evidence that GFI can impact the input-output efficiency of traditional hospitals and promote its development. In this regard, the governments can decide and carefully schedule the evaluation period. Our findings can provide new evidence and decision-making basis for the government to develop policies to promote the development of traditional medicine and increase the supply of traditional medicine services.

## Data Availability

The data that support the findings of this study are available from Health Commission of Henan Province, but restrictions apply to the availability of these data, which were used under license for the current study, and so are not publicly available. Data are however available from the authors upon reasonable request and with permission of Health Commission of Henan Province.

## Abbreviations

TCM: Traditional Chinese Medicine
GFI: Governmental financial investment
PTHs: Public TCM hospitals
SFA: Stochastic Frontier Analysis
BEG: Barro Economic Growth
Revenue: The revenue of PTHs
Clinical Workers: Number of TCM practicing clinical workers
Fixed Assets: Value of fixed assets
Technologies: Number of suitable TCM technologies implemented
Improvement Rate: TCM treatment improvement rate
MPG: Marginal Product of Government
DEA: Data Envelopment Analysis
ADF: Augmented Dickey-Fuller
